# DNA methylation-based surrogate markers of C-reactive protein and their associations with health-related traits

**DOI:** 10.1007/s11357-025-02085-8

**Published:** 2026-01-22

**Authors:** Danmeng Lily Li, Allison M. Hodge, Joanne Ryan, Melissa C. Southey, Graham G. Giles, Roger L. Milne, Pierre-Antoine Dugué

**Affiliations:** 1 Precision Medicine, School of Clinical Sciences at Monash Health, Monash University, Clayton, VIC Australia; 2 Cancer Epidemiology Division, Cancer Council Victoria, Melbourne, VIC Australia; 3 Centre for Epidemiology and Biostatistics, Melbourne School of Population and Global Health, The University of Melbourne, Parkville, VIC Australia; 4 School of Public Health and Preventive Medicine, Monash University, Melbourne, VIC Australia; 5 Department of Clinical Pathology, The University of Melbourne, Parkville, VIC Australia

**Keywords:** Low-grade chronic inflammation, CRP, DNA methylation, Biological ageing

## Abstract

Several methylation-based surrogate markers of C-reactive protein (*mCRP*) have been proposed and used to assess the risk of age-related phenotypes and construct novel ageing markers. We aimed to (i) assess the variance in plasma CRP explained by several *mCRP* markers; (ii) compare their associations with three health-related traits: mortality, body mass index (BMI), and *PCGrimAge*; and (iii) assess the stability of CRP and *mCRP* over a decade. Blood samples were collected from 947 participants in the Melbourne Collaborative Cohort Study at baseline (1990–1994) and wave 2 (2003–2007). High-sensitivity CRP was measured in plasma samples. Five *mCRP* markers were calculated: *mCRP-Ligthart*, *mCRP-Wielscher*, *mCRP-EpiScore*, *mCRP-GrimAge2*, and *mCRP-Hillary*. Intraclass correlation coefficients (ICCs) were calculated to assess the stability of CRP/*mCRP* between baseline and wave 2. Associations of wave 2 CRP/*mCRP* with mortality (*N*_deaths_ = 319) were assessed using Cox models and linear regression for BMI and age-adjusted *PCGrimAge*. *mCRP* explained 6.0% (*mCRP-Wielscher*) to 16.8% (*mCRP-GrimAge2*) of the variance in plasma CRP. The ICCs for CRP and *mCRP* markers were similar, ranging from 0.44 (*mCRP-GrimAge2*) to 0.63 (*mCRP-Wielscher*). Compared with CRP, *mCRP* had stronger associations with *PCGrimAge*, whereas for mortality and BMI, the associations were similar or weaker for *mCRP*. Associations between *mCRP* and mortality were greatly attenuated after adjusting for *PCGrimAge* but less so after adjusting for CRP. The association of CRP with mortality remained strong after adjusting for *mCRP*. Our study validated *mCRP* markers and clarified the role of CRP and *mCRP* in ageing, which may inform the use and development of ageing biomarkers.

**Supplementary Information:**

The online version contains supplementary material available at https://doi.org/10.1007/s11357-025-02085-8.

## Introduction

Inflammation is the process by which the immune system recognises and protects the host from harmful or foreign stimuli [1]. Low-grade chronic inflammation is one of the hallmarks of ageing [2]. Inflammatory biomarkers such as C-reactive protein (CRP), interleukin-6 (IL-6), and numerous cytokines have been widely studied for their roles in ageing and age-related phenotypes [2–4]. However, as an acute-phase protein, CRP is prone to rapid elevation in response to infection, inflammation, drug intake, and other health events [5]. Considerable fluctuations in CRP levels have been reported by previous epidemiological studies [6, 7], limiting the utility of CRP as a stable marker of age-related chronic health conditions.

Blood DNA methylation (DNAm), one of the most-studied epigenetic processes that involves the addition of a methyl group to the DNA, is considered to (i) have short-term biological stability [8, 9], (ii) regulate gene expression, and (iii) be sensitive to exogenous and endogenous exposures accumulated over the lifetime [10, 11]. This has prompted the development of surrogate markers of lifestyle factors [12, 13], inflammation [14–18], ageing [19–22], and disease risks [23]. These methylation-based predictors are considered to provide additional information beyond their targeted traits and DNAm [24].

A number of methylation-based surrogate markers of CRP (*mCRP*) have been developed [14–17, 19] based on large epigenome-wide association studies (EWAS). It has also been reported that *mCRP* may be more strongly associated with health-related traits than measured CRP [25–28]. Three studies [9, 25, 26] reported that compared with plasma CRP, one such methylation marker *mCRP-Ligthart* [14] had weaker associations with body mass index (BMI) and depression, but stronger associations with cognitive function and brain health. Stevenson et al. [9] reported a higher intraclass correlation coefficient (ICC) over four waves (9 years) of follow-up for *mCRP-Ligthart* than for plasma CRP, indicating greater temporal stability of *mCRP*. Verschoor et al. [27] assessed *mCRP-Wielscher* [15] and reported that compared with serum CRP, the associations for *mCRP* were stronger with risks of cardiovascular disease (CVD) and asthma, but weaker with all-cause mortality and BMI. A recent study reported that *mCRP-Hillary* [17] showed stronger associations with risks of diabetes and obstructive sleep apnoea-related traits than plasma CRP [28]. Existing evidence mainly compared plasma CRP with *mCRP* based on EWAS by Ligthart et al. [14] or Wielscher et al. [14], from which CpGs were selected based on results from linear or linear mixed models. Two studies [17, 28] assessed more up-to-date markers [16, 17, 19]. Evidence on broader comparisons across different *mCRP* in an external cohort is lacking.

In this study, we considered five *mCRP* markers [14–17, 19] in a cohort of middle-aged and older Australians and aimed to (i) assess the performance of each *mCRP* marker in predicting plasma CRP, (ii) compare the associations of *mCRP* and plasma CRP with health-related traits and explore their overlap, and (iii) assess the temporal stability of plasma CRP and *mCRP* over a decade.

## Methods

### Study sample

This study used data from the Melbourne Collaborative Cohort Study (MCCS), a prospective study that recruited 41,513 middle-aged and older Australians (99% aged between 40 and 69 years, 59% women) of white European origin between 1990 and 1994 (baseline). During 2003–2007, a face-to-face follow-up (wave 2) was carried out. At both waves, socio-demographic, lifestyle, and health data, as well as physical measurements and blood samples were collected [29].

#### Ethics and consent to participate

The MCCS was approved by the Human Research Ethics Committee of Cancer Council Victoria, Melbourne, Victoria, Australia, and informed consent was provided by all participants according to the Declaration of Helsinki.

### CRP measurement

This study used a subset of 1100 controls from six cancer case-control studies nested in the MCCS, where participants had their peripheral blood samples collected at baseline and wave 2, from which plasma samples were extracted and stored in liquid nitrogen at − 180 °C until transportation for laboratory analysis. Plasma CRP (µg/mL) was assayed at baseline and wave 2 using a highly sensitive matrix-assisted laser desorption ionisation-time of flight mass spectrometry (MALDI-TOF MS) assay at Bevital laboratory in Bergen, Norway http://www.bevital.no/ [30]. It was log-transformed as ln(CRP + 1) and standardised to a mean of zero and standard deviation of one in all analyses.

### DNA methylation markers of CRP (mCRP)

At baseline and wave 2, these peripheral blood samples were stored as dried blood spots on Guthrie cards. Genome-wide DNAm was measured using the Illumina HumanMethylation450 BeadChip array [29], and underwent standard normalisation and quality control procedures [31].

Five *mCRP* markers (Table 1) were calculated: *mCRP-Ligthart*, *mCRP-Wielscher*, *mCRP-EpiScore*, *mCRP-GrimAge2*, and *mCRP-Hillary*. *mCRP-Ligthart* was calculated based on eight CpGs that were associated with CRP in a large EWAS, using as weights the beta coefficients of association with CRP extracted from the original publication [14]. The 68-CpG *mCRP-Wielscher* was calculated based on 68 out of 1511 CpGs identified by Wielscher et al. [15] and differentially methylated in CRP-treated cells in Verschoor et al.’s [27] in vitro experiment, using Wielscher et al.’s [15] EWAS results as weights. *mCRP-EpiScore* was based on 100 CpGs selected via elastic net regression by Gadd et al. [16] and calculated using the *methscore* function in R [32]. *mCRP-GrimAge2* was developed by regressing log2(CRP) on age, sex, and the 1030 CpGs included in *GrimAge* [19] and using elastic net regression, which selected 132 CpGs; it was calculated using R code provided by the authors. From three *mCRP* markers developed by Hillary et al. [17], we chose the one developed with elastic net regression, as the authors reported that it performed better than the others and recommended it. *mCRP-Hillary* was calculated based on the selected 1468 CpGs using the weights provided in the original article [17].

**Table 1:** Description of five previously developed methylation-based markers of CRP

*mCRP*	*N* of CpGs	Development	Training data	Testing data	Correlation with CRP	Notes
***mCRP-Ligthart*** (2016) [14]	8	Linear mixed model: adjusted for age, sex, WBC proportion, technical covariates, BMI and smoking	11 cohorts (European ancestry), *N* = 8863	5 cohorts (African American), *N* = 4111 (replication set)	0.15–0.24	8/58 replicated CpGs that were independent of each other were selected for mCRP-Ligthart
***mCRP-Wielscher*** (2022) [15]	68 (1511)	Linear model: adjusted for age, sex, WBC proportion and technical covariates	30 cohorts, *N* = 22,774	NA	NA	68/1511 CpGs were further selected by Verschoor et al. [27] as being differentially methylated in CRP vs mock-treated cells in their in vitro experiment. The new mCRP exhibited a stronger association with age-related traits [27]
***mCRP-EpiScore*** (2022) [16]	100	Elastic net regression model: adjusted for age, sex, 20 genetic principal components and pQTLs of CRP	KORA, *N* = 994	Generation Scotland, *N* = 778	0.29	Included in bAge [22]
***mCRP-GrimAge2*** (2022) [19]	132	Elastic net regression model: lnCRP regressed on age, sex and the 1030 CpGs in GrimAge	FHS Offspring, *N* = 1833	FHS offspring *N* = 711	0.48	Included in GrimAge2
***mCRP-Hillary*** (2024) [17]	1468	Elastic net regression model	Generation Scotland, *N* = 17,936	Validated in 5 cohorts separately	0.27–0.53	3 mCRP markers were developed using (i) elastic net regression, (ii) Bayesian penalised regression, and (iii) principal-component analysis and elastic net regression [17]. The mCRP marker derived from elastic net regression only (first method) performed better in predicting CRP than the other 2 [17]

*CRP* C-reactive protein, *WBC* white blood cell proportion, *KORA* Cooperative Health Research in the region of Augsburg, *FHS* Framingham Heart Study

All *mCRP* markers were standardised to a mean of zero and a standard deviation of one to be comparable.

### Health-related traits

Three health-related traits were considered: all-cause mortality, body mass index (BMI, kg/m^2^) and *PCGrimAge*. All-cause mortality is a widely studied ageing hard endpoint. Participants were passively followed from the time they attended the wave 2 interview to 29th February 2020. Deaths were identified via annual record linkage to the Victorian Registry of Births, Deaths, and Marriages [29]. BMI is a widely used indicator of overall body size, the development of which shares similar hallmarks with ageing, including inflammation, metabolic dysfunction, insulin resistance, and impaired immune function [33]. As a major aspect of obesity development, adipocyte enlargement contributes to the secretions of cytokines and adipokines that are responsible for chronic low-grade inflammation [33]. Physical measurement of weight was performed by trained personnel at wave 2, and height was taken from baseline measurement to calculate BMI [29]. *PCGrimAge* is a powerful, recently developed epigenetic marker of biological ageing that used principal component analyses to select CpGs for *GrimAge* to improve its reliability and does not include a surrogate marker of CRP [20, 21]. It was reported to have strong associations with various age-related health events [34, 35]. Therefore, we used it to represent biological ageing primarily reflected in epigenetic processes rather than inflammation. *PCGrimAge* was calculated using the *methscore* function in R [32].

### Data preparation

After excluding participants with missing plasma CRP data (*n* = 29) at wave 2, 947 participants were available for the analysis. A small proportion of missing data in covariates (0.1–10.7%, Table S1) was imputed using the *missForest* method, which performs well with mixed data types and a small proportion of missing data [36].

### Statistical analyses

We used wave 2 data for the main analyses to avoid survivor bias in analyses of mortality since participants included in the analyses had to be alive at least until wave 2.

Pearson correlations were calculated between CRP (log-transformed) and five *mCRP* markers, as well as demographic and lifestyle-related covariates collected at wave 2 including age, smoking pack-years (log-transformed), alcohol consumption (grammes/day, Supplementary Methods), BMI, diet quality (Alternative Healthy Eating Index 2010 [AHEI-2010] [37], calculated from food frequency questionnaire data), physical activity (metabolic equivalent of task hours/week), total cholesterol concentration (mmol/L), and *PCGrimAge* (adjusted for age).

We used Cox proportional hazard models to assess the individual associations of wave 2 CRP and *mCRP* with mortality over up to 17 years. Five models were considered: Model 0 was unadjusted; Model 1 adjusted for age at blood draw, sex, and country of birth (Australia/New Zealand, Northern Europe, Southern Europe); Model 2 additionally adjusted for socio-demographic and lifestyle-related covariates including socioeconomic status (measured by the socioeconomic indexes for areas, decile), education level (1–8), smoking status (current, former, never smokers), smoking pack-years, alcohol consumption, physical activity, BMI, and AHEI-2010; Model 3 additionally adjusted for white blood cell (WBC) proportions (including CD8 + T cell, CD4 + T cell, natural killer cell, B cell, and monocyte); Model 4 included all CRP/*mCRP* markers and adjusted for age, sex, and country of birth to assess the extent to which the association of plasma CRP with mortality could be explained by all *mCRP* markers collectively. Model 5 adjusted for *PCGrimAge*, or *mCRP* marker or CRP respectively in addition to Model 1*.* A restricted cubic spline term (3 knots at the 10th, 50th, and 90th percentiles) was applied to alcohol consumption and BMI to account for their potentially non-linear associations with health-related phenotypes [38, 39]. The Harrell’s concordance index (c-index) was used to assess the contribution of CRP, *mCRP*, and *PCGrimAge*, and their combinations to mortality prediction and further explore their overlap and complementarity. Linear regression models were fitted with the same covariates as in Models 1, 2, 3, and 5 to assess the associations of CRP and *mCRP* with BMI and *PCGrimAge* (used as exposures and outcomes, respectively).

To assess the stability of CRP and *mCRP*, we first used univariate linear regression to assess the associations between changes in CRP, *mCRP*, and *PCGrimAge* over follow-up and time between baseline and wave 2 visits. We then calculated the two-way fixed-effect ICCs and inter-wave correlation coefficients for age-adjusted CRP and *mCRP* at baseline and wave 2 using the 899 participants who had CRP data at both time points. Bland–Altman plots were presented to show the distribution of changes in CRP and *mCRP* between baseline and wave 2.

### Sensitivity analysis

To assess the robustness of the findings to the potential presence of acute-phase inflammation, we repeated the analyses of mortality associations for participants with CRP levels below 10 µg/mL (*N* = 907) as per recommendations from large population-based studies [40].

All analyses were conducted with R 4.4.1.

## Results

### Sample characteristics

Of 947 participants at wave 2, 69% were males, Table S2. The mean age at blood draw was 69 years. Participants were, on average, followed for 13 years, and there were 319 deaths. The average CRP concentration was 2.23 µg/mL in the entire sample and was slightly higher (3.22 µg/mL) for those who died during follow-up.

**Fig. 1:**
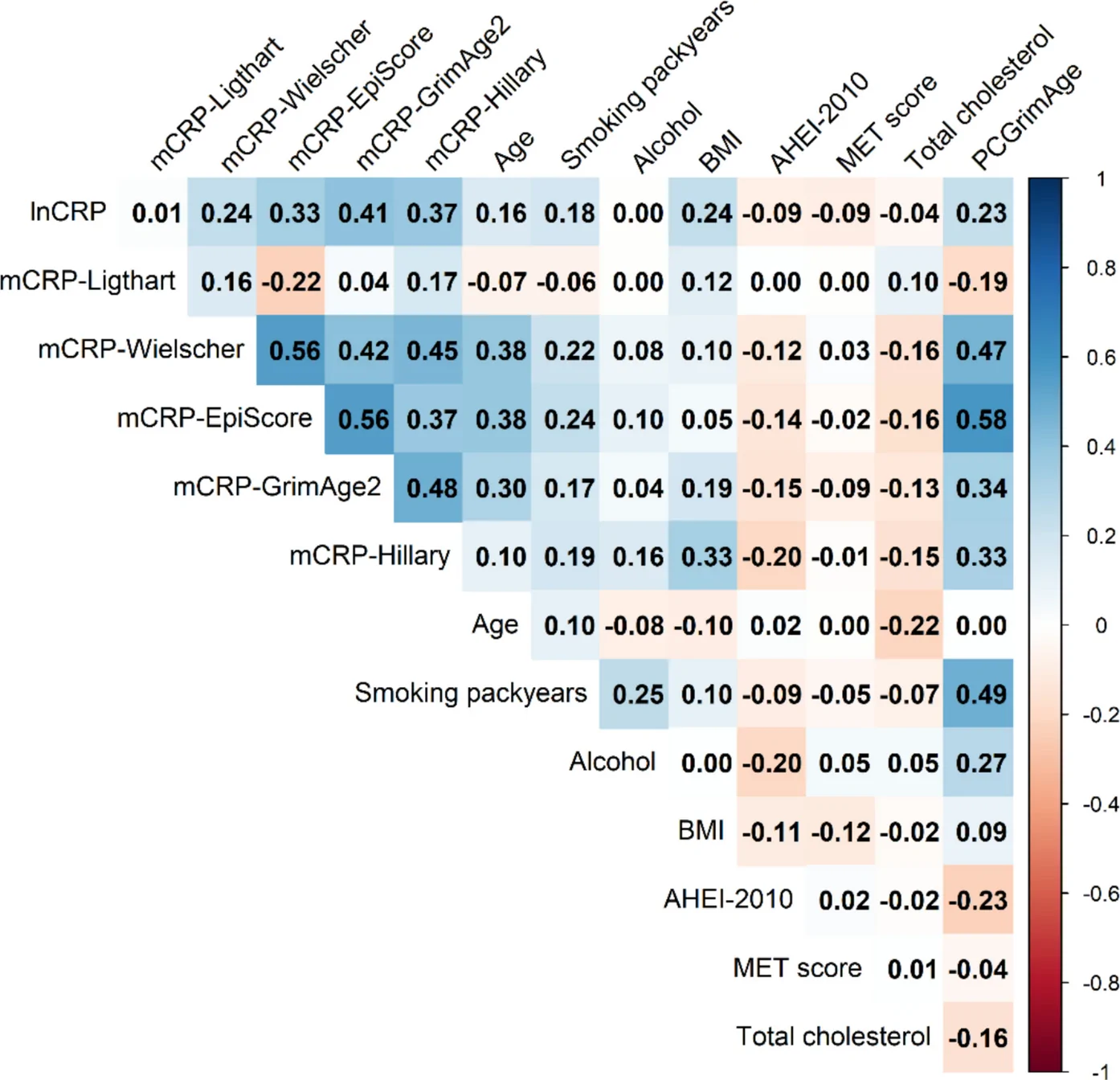
Pearson correlation matrix of plasma hsCRP, *mCRP*, and lifestyle factors; BMI, body mass index; AHEI-2010, Alternative Healthy Eating Index 2010; MET, metabolic equivalent of task; hsCRP, highly sensitive C-reactive protein. hsCRP and smoking pack-years were log-transformed as ln(CRP + 1)/ln(pack-years + 1). The *PCGrimAge* was age-adjusted

Plasma CRP (log-transformed) and *mCRP* showed generally weak to moderate correlation with lifestyle factors and total cholesterol concentration. The correlations were slightly weaker for CRP than for *mCRP* markers, except for physical activity (*r* =  − 0.09, Fig. 1). *mCRP-EpiScore* showed moderate correlations with age-adjusted *PCGrimAge* (*r* = 0.58) and age (*r* = 0.38).

### Correlations between CRP and mCRP

With the exception of *mCRP-Ligthart*, which showed no correlation with CRP (*r* = 0.01), the other *mCRP* markers showed moderate correlations with CRP, ranging from 0.24 (*mCRP-Wielscher*) to 0.41 (*mCRP-GrimAge2*), Fig. 1. Stronger correlations were observed between *mCRP* markers (e.g., *mCRP-EpiScore*/*mCRP-Wielscher*: *r* = 0.56). The variance in CRP explained by *mCRP* ranged between 6.0% for *mCRP-Wielscher* and 16.8% for *mCRP-GrimAge2*.

### Plasma CRP, mCRP, and mortality

In Model 1, an SD increase in CRP was associated with a 1.31-fold (95% CI, 1.18–1.44; Table 2) higher mortality risk. The HR was slightly higher for *mCRP-EpiScore* (HR = 1.38; 95% CI, 1.22–1.55), but lower for other *mCRP* (HRs range from 1.15 for *mCRP-Hillary* to 1.26 for *mCRP-Wielscher*) and null for *mCRP-Ligthart*. Minimal attenuations were observed for all markers after adjusting for sociodemographic and lifestyle factors (Model 2), although the HR became non-significant for *mCRP-Hillary*. Associations after adjustment for WBC proportions (Model 3) remained similar for all markers except *mCRP-Ligthart*, for which the HR increased to 1.15 (95% CI, 0.99–1.34).

**Table 2:** The associations of plasma hsCRP and *mCRP* with health-related traits (*N* = 947)

Health-related traits		**hsCRP**			***mCRP-Lighart***			***mCRP-Wielscher***		
		HR/*β*	95% CI	*P*	HR/*β*	95% CI	*P*	HR/*β*	95% CI	*P*
Mortality (*N*_deaths_ = 319)	Model 0^a^	1.28	1.16, 1.41	**1 × 10**^**−6**^	0.99	0.89, 1.11	0.87	1.22	1.09, 1.37	**8 × 10**^**−4**^
	Model 1^b^	1.31	1.18, 1.44	**1 × 10**^**−7**^	1.02	0.91, 1.14	0.77	1.26	1.11, 1.42	**2 × 10**^**−4**^
	Model 2^c^	1.27	1.14, 1.40	**9 × 10**^**−6**^	1.00	0.89, 1.12	0.99	1.19	1.05, 1.35	**7 × 10**^**−3**^
	Model 3^d^	1.25	1.12, 1.39	**5 × 10**^**−5**^	1.15	0.99, 1.34	0.07	1.26	1.09, 1.47	**2 × 10**^**−3**^
	Model 4^e^	1.24	1.11, 1.39	**1 × 10**^**−4**^	1.05	0.93, 1.20	0.44	1.12	0.96, 1.31	0.15
BMI (exposure)	Model 0	0.24	0.18, 0.31	**3 × 10**^**−14**^	0.12	0.06, 0.18	**2 × 10**^**−4**^	0.10	0.03, 0.16	**3 × 10**^**−3**^
	Model 1	0.24	0.18, 0.31	**4 × 10**^**−14**^	0.11	0.05, 0.17	**8 × 10**^**−4**^	0.12	0.06, 0.17	**2 × 10**^**−4**^
	Model 2	0.22	0.15, 0.28	**2 × 10**^**−11**^	0.12	0.05, 0.18	**6 × 10**^**−4**^	0.10	0.04, 0.16	**1 × 10**^**−3**^
	Model 3	0.23	0.17, 0.29	**1 × 10**^**−12**^	0.06	0.01, 0.11	**0.01**	0.10	0.05, 0.15	**8 × 10**^**−5**^
*PCGrimAge*	Model 0	0.23	0.17, 0.29	**6 × 10**^**−13**^	− 0.19	− 0.25, − 0.13	**5 × 10**^**−9**^	0.47	0.41, 0.52	**8 × 10**^**−53**^
	Model 1	0.25	0.19, 0.30	**2 × 10**^**−16**^	− 0.14	− 0.20, − 0.08	**4 × 10**^**−6**^	0.50	0.45, 0.56	**9 × 10**^**−62**^
	Model 2	0.17	0.11, 0.22	**5 × 10**^**−10**^	− 0.14	− 0.19, − 0.09	**2 × 10**^**−8**^	0.41	0.36, 0.46	**2 × 10**^**−53**^
	Model 3	0.11	0.06, 0.16	**2 × 10**^**−5**^	0.09	0.03, 0.15	**3 × 10**^**−3**^	0.51	0.46, 0.56	1** × 10**^**−74**^
Health-related traits		***mCRP-EpiScore***			***mCRP-GrimAge2***			***mCRP-Hillary***		
		HR/*β*	95% CI	*P*	HR/*β*	95% CI	*P*	HR/*β*	95% CI	*P*
Mortality (*N*_deaths_ = 319)	Model 0	1.34	1.19–1.50	**1 × 10**^**−6**^	1.21	1.07–1.36	**2 × 10**^**−3**^	1.16	1.03–1.29	**0.01**
	Model 1	1.38	1.22–1.55	**9 × 10**^**−8**^	1.25	1.11–1.41	**3 × 10**^**−4**^	1.15	1.03–1.29	**0.02**
	Model 2	1.31	1.16–1.48	**2 × 10**^**−5**^	1.19	1.05–1.34	**8 × 10**^**−3**^	1.10	0.97–1.24	0.14
	Model 3	1.28	1.11, 1.47	**5 × 10**^**−4**^	1.15	1.00, 1.31	**0.04**	1.09	0.96, 1.24	0.16
	Model 4	1.26	1.09, 1.45	**1 × 10**^**−3**^	1.13	0.99, 1.29	0.07	1.08	0.95, 1.23	0.22
BMI (exposure)	Model 0	0.05	− 0.01, 0.11	0.13	0.19	0.13, 0.25	**6 × 10**^**−9**^	0.33	0.27, 0.39	**8 × 10**^**−26**^
	Model 1	0.08	0.02, 0.15	**6 × 10**^**−3**^	0.22	0.16, 0.28	**2 × 10**^**−12**^	0.33	0.27, 0.40	**8 × 10**^**−26**^
	Model 2	0.06	0.00, 0.12	**0.04**	0.19	0.13, 0.25	**5 × 10**^**−10**^	0.32	0.26, 0.38	**4 × 10**^**−24**^
	Model 3	0.10	0.05, 0.16	1** × 10**^**−4**^	0.22	0.16, 0.28	7** × 10**^**−14**^	0.33	0.27, 0.39	1** × 10**^**−24**^
*PCGrimAge*	Model 0	0.58	0.53, 0.63	**1 × 10**^**−86**^	0.34	0.28, 0.40	**7 × 10**^**−27**^	0.33	0.27, 0.39	**5 × 10**^**−25**^
	Model 1	0.67	0.63, 0.72	**7 × 10**^**−131**^	0.42	0.37, 0.48	**1 × 10**^**−45**^	0.27	0.21, 0.33	**1 × 10**^**−19**^
	Model 2	0.58	0.54, 0.62	**5 × 10**^**−122**^	0.33	0.28, 0.38	**2 × 10**^**−34**^	0.19	0.13, 0.24	**1 × 10**^**−11**^
	Model 3	0.53	0.48, 0.58	4** × 10**^**−86**^	0.24	0.18, 0.29	1** × 10**^**−18**^	0.18	0.13, 0.23	3** × 10**^**−12**^

hsCRP was log-transformed as ln(CRP + 1). hsCRP, *mCRP*, *PCGrimAge*, and BMI were standardised to a mean of 0 and a standard deviation of 1

*hsCRP* highly sensitive C-reactive protein

^a^Model 0 is unadjusted

^b^Model 1 is adjusted for age, sex, and country of birth

^c^Model 2 is adjusted for age, sex, country of birth, SEIFA-10, education levels, smoking status, smoking pack-years, alcohol consumption (spline), AHEI-2010, physical activity, and BMI (spline)

^d^Model 3 is adjusted for age, sex, country of birth, SEIFA-10, education levels, smoking status, smoking pack-years, alcohol consumption (spline), AHEI-2010, physical activity, BMI (spline), and white blood cell proportion (including CD8 + T cell, CD4 + T cell, natural killer cell, B cell, and monocyte)

^e^Model 4 included age, sex, country of birth, plasma hsCRP, and all five *mCRP* markers

**Fig. 2:**
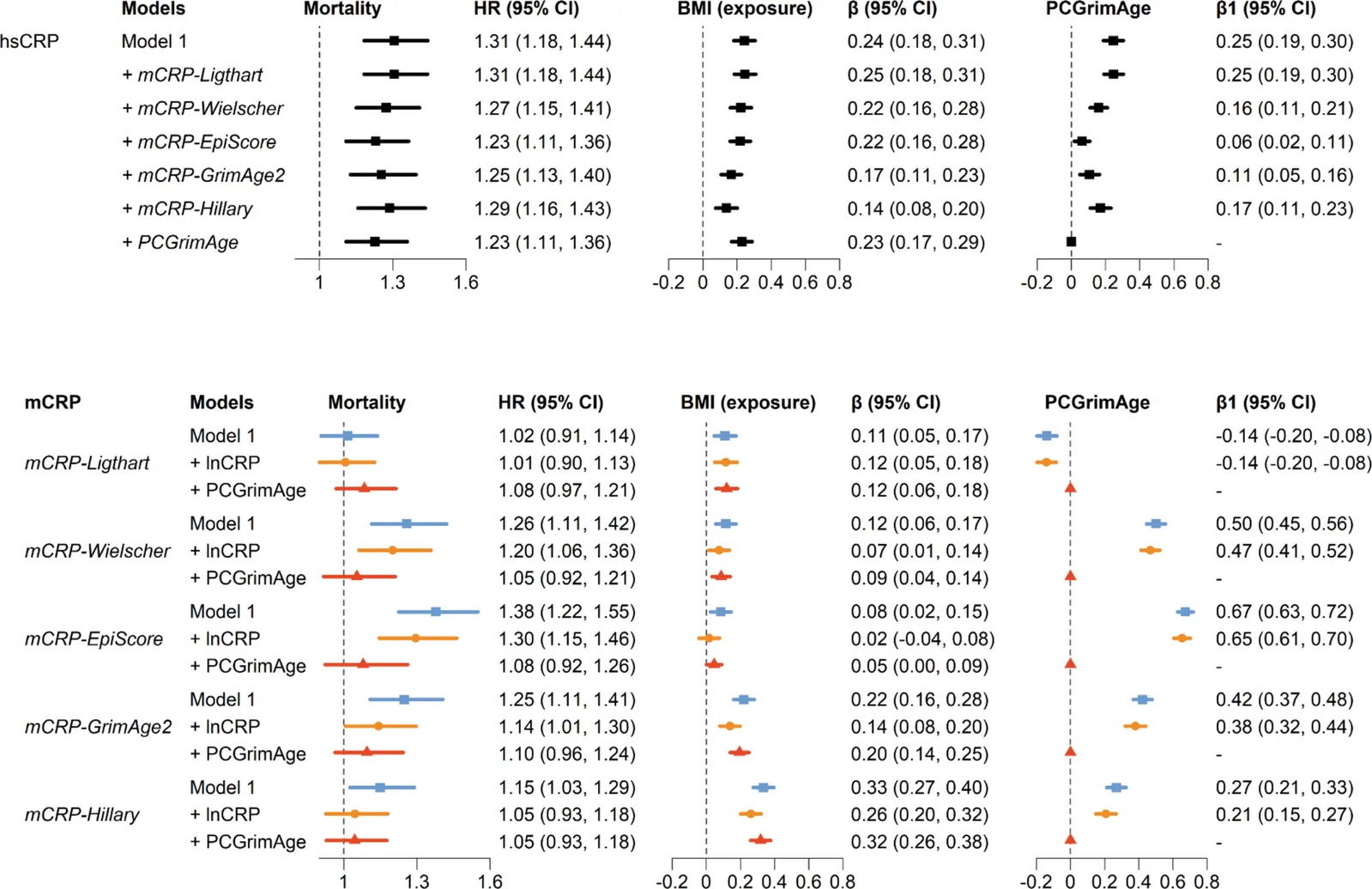
Associations of CRP and *mCRP* with three health-related traits (mortality (outcome), BMI (exposure), and *PCGrimAge* (outcome)) (*N* = 947). Model 1 was adjusted for age, sex, and country of birth; the other models added the marker specified to Model 1; hsCRP, highly sensitivity C-reactive protein. CRP was log-transformed as ln(CRP + 1). The *PCGrimAge* was age-adjusted. The *PCGrimAge*, BMI, CRP, and *mCRP* markers were standardised to a mean of 0 and standard deviation of 1

After adjusting for *PCGrimAge*, CRP retained a strong association with mortality (HR = 1.23; 95% CI, 1.11–1.36; Fig. 2), whereas the associations for *mCRP* were all close to null (HRs from 1.05 to 1.10). Only minimal attenuations were observed after adjusting CRP for all (Model 4, Table 2) or any (Model 5, Fig. 2) *mCRP*, e.g., Model 4, adjusting for all five *mCRP*: HR = 1.24; 95% CI, 1.11–1.39. The associations for *mCRP-EpiScore* remained strong after accounting for CRP (HR = 1.30; 95% CI, 1.15–1.46). Only the association of *mCRP-Hillary* with mortality attenuated close to null after adjusting for CRP: HR = 1.05, 95% CI 0.93–1.18.

In the univariate models, among all *mCRP* markers, *mCRP-EpiScore* showed the highest c-index of 0.656, Table S3. The c-index for CRP (*c* = 0.595) was higher than that of *mCRP-Hillary* (*c* = 0.564), but lower than that for other *mCRP* (the *c* range is between 0.618 for *mCRP-GrimAge2* and 0.656 for *mCRP-EpiScore*). After adjusting for age, sex, and country of birth (*c* = 0.742), plasma CRP (*c* = 0.753), *mCRP-EpiScore* (*c* = 0.754), and *PCGrimAge* (*c* = 0.762) showed the greatest increase in mortality prediction. The c-index for the model including CRP and *PCGrimAge* was 0.765, which was greater than the model including all CRP/*mCRP* markers (*c* = 0.759) or *PCGrimAge* and all *mCRP* markers (*c* = 0.763). The model with all CRP/*mCRP* markers and *PCGrimAge* had a c-index of 0.766.

### Sensitivity analysis

Excluding participants with high CRP levels (> 10 µg/mL) slightly attenuated the associations of CRP and all *mCRP* with mortality, Table S4. One SD increase in CRP was associated with 1.27-fold (95% CI, 1.13–1.42) higher mortality risk. This association was slightly stronger for *mCRP-EpiScore*: HR = 1.34, 95% CI, 1.18–1.52, and was close to null for *mCRP-Hillary*: HR = 1.09; 95% CI, 0.97–1.23.

### Plasma CRP, mCRP, and BMI

BMI was positively associated with CRP and *mCRP* levels. Similar associations were observed for CRP (Model 1: per SD, *β* = 0.24; 95% CI, 0.18–0.31; Table 2) and *mCRP-GrimAge2* (*β* = 0.22; 95% CI, 0.16–0.28). These were stronger for *mCRP-Hillary* (*β* = 0.33) and close to null for *mCRP-EpiScore* (*β* = 0.08). After adjusting for WBC proportions, the associations remained strong and positive (*β* ranging from 0.10 for *mCRP-Wielscher* and *mCRP-EpiScore* to 0.33 for *mCRP-Hillary*), with *mCRP-Ligthart* showing a greater relative decrease from *β* = 0.12 to 0.06. The association of BMI with CRP was greatly attenuated after the adjustment for *mCRP-Hillary* (*β* = 0.14), Fig. 2, more so than that for other *mCRP* markers. For the associations of BMI with *mCRP*, adjusting for CRP resulted in stronger attenuations, particularly for *mCRP-GrimAge2* (*β* from 0.22 to 0.14), whereas adjusting for *PCGrimAge* led to marginal attenuations.

### Plasma CRP, mCRP, and PCGrimAge

Similar associations with *PCGrimAge* were observed for CRP and *mCRP-Hillary*, where one SD increase was associated with 0.17-SD (95% CI, 0.11–0.22; Table 2) and 0.19-SD (95% CI, 0.13–0.24) higher *PCGrimAge*, respectively. The associations for *mCRP-Wielscher*, *mCRP-EpiScore*, and *mCRP-GrimAge2* were much stronger, particularly for *mCRP-EpiScore* (*β* = 0.67). After adjusting for WBC proportions, the effect estimate was reversed for *mCRP-Ligthart* (*β* =  − 0.14 in Model 2 to 0.09 in Model 3). Adjusting for CRP only led to minimal attenuations, Fig. 2.

### Stability of CRP and mCRP

**Fig. 3:**
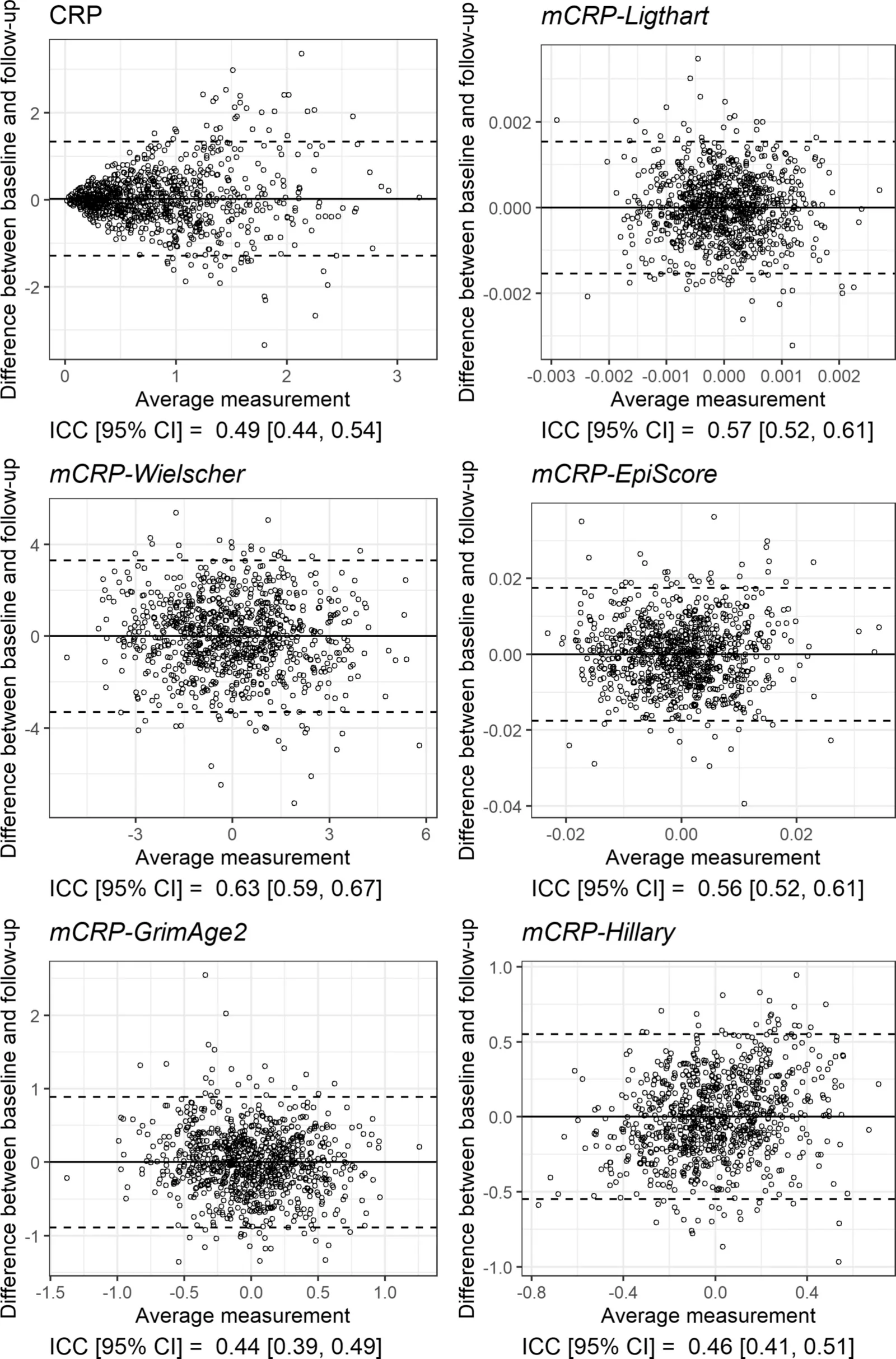
Bland–Altman plots for plasma hsCRP and *mCRP* at baseline and wave 2 (*N* = 899). ICCs were calculated using the *irr* package in R, using two-way ANOVA, with fixed effect of follow-up waves being considered, and individual measurements as the unit of reliability. The ICCs are therefore measuring consistency of the measures (CRP/*mCRP*) over follow-up of a decade; hsCRP, highly sensitive C-reactive protein. hsCRP was log-transformed as ln(CRP + 1). hsCRP and *mCRP* were all adjusted for age

There was a substantial increase in *PCGrimAge* (*β* = 0.43 SD change, *P* = 3 × 10^−60^) during follow-up. Weaker, but notable increases were also observed in *mCRP-EpiScore* (*β* = 0.13, *P* = 3 × 10^−6^) and *mCRP-*GrimAge2 (*β* = 0.11, *P* = 2 × 10^−4^) from baseline to wave 2, less so for *mCRP-Hillary* (*β* = 0.05, *P* = 0.08), Table S5. There were minimal changes in CRP, *mCRP*-*Ligthart*, and *mCRP*-*Wielscher* (*P* range, 0.13–0.28). We found strong correlations for all baseline markers with their wave 2 measurements, 0.49 for CRP, and ranging from 0.47 for *mCRP-GrimAge2* to 0.67 for *mCRP-Wielscher*, Table S5. The ICC for CRP over time points was 0.48. It ranged between 0.44 for *mCRP-GrimAge2* and 0.63 for *mCRP-Wielscher*, Fig. 3. In the Bland–Altman plots, more variability in CRP over follow-up was found at higher CRP concentrations, which was not observed for *mCRP* markers.

## Discussion

Our study of five *mCRP* markers may help to clarify the role of CRP and *mCRP* in ageing and inform the future development of ageing biomarkers. We found that *mCRP* markers showed moderate correlation with plasma CRP. The temporal stability of *mCRP* was marker-dependent and not generally greater than that of CRP. Lower concentrations of CRP showed less variation over time than did high concentrations, which was not observed for any *mCRP* markers. *mCRP* markers were associated with mortality risk, with similar effect sizes to plasma CRP. For all *mCRP* markers, the associations with mortality were close to null after accounting for *PCGrimAge*, and this was not observed for plasma CRP. Compared with plasma CRP, *mCRP* markers generally showed stronger associations with *PCGrimAge* and more variable associations with BMI. The associations of *mCRP* with BMI were partially explained by CRP, but not by *PCGrimAge*. Including WBC proportions had heterogeneous but limited influence on the associations, except with regard to *mCRP-Ligthart*.

To our knowledge, this is the first study to provide a comprehensive external validation and comparison of five widely used *mCRP* markers. Most markers were originally tested in external cohorts and showed correlations with measured CRP in the same range as our study, from 0.29 for *mCRP-EpiScore* to 0.27–0.53 for *mCRP-Hillary* [14, 16, 17, 19]. The associations of CRP and *mCRP* with health-related traits were similar to previous findings as well. Verschoor et al. [27] assessed *mCRP-Wielscher* in 1446 participants from the Canadian Longitudinal Study on Aging and reported stronger associations with epigenetic ageing and other health outcomes such as asthma and CVDs but weaker associations with mortality and BMI, compared to plasma CRP. Hillary et al. [17] found that *mCRP-Hillary* was more strongly associated with BMI, diabetes, and CVDs than CRP, but equally strongly associated with all-cause mortality.

Plasma CRP is usually considered to be reasonably stable in the short term but less so in the long term [6, 7, 41], whereas the stability of DNA methylation is CpG-dependent [42]. Stevenson et al. [9] reported a higher ICC (0.82 for *mCRP* vs 0.72 for CRP) for the 7-CpG *mCRP* derived from Ligthart’s EWAS compared with plasma CRP over four waves of follow-up (a 3-year interval). In our study, the ICCs of CRP and *mCRP* measurements over a decade were generally lower. The temporal stability of *mCRP* was not generally greater than that of CRP, particularly for *mCRP-GrimAge2* and *mCRP-Hillary*. In addition, the positive associations of changes in elastic net-derived *mCRP* markers, particularly *mCRP-EpiScore* and *mCRP-GrimAge2*, with follow-up time, although weaker than *PCGrimAge*, suggested their potential application in future ageing studies.

The unique, divergent distribution of changes in CRP over a decade aligns with the fact that lower CRP levels, usually referred to as normal or low-grade inflammation, tend to remain stable, whereas greater variations in CRP might indicate more acute, temporary conditions such as infection or trauma [5] at or close to blood draw. This pattern was not observed for any of the *mCRP* markers, for which larger changes in the marker between baseline and wave 2 were evenly distributed across participants’ average values. This might indicate that *mCRP* markers may, to some extent, be less responsive to certain acute health conditions, which would allow for more reliable assessments of chronic low-grade inflammation. A previous study based in the MCCS showed that inflammatory biomarkers were largely independent of epigenetic age [43], and in our study, there was also little overlap in the associations of CRP and *mCRP* with mortality. These findings are consistent with the fact that although chronic inflammation can occur subsequent to age-related epigenetic alterations, it remains an independent hallmark of ageing [44].

Our results suggest that *mCRP* did not fully capture measured CRP and tended to show different properties representing the crossover of inflammation- and methylation-related aspects of biological ageing (Table S6). *mCRP-Wielscher* showed the highest temporal stability among all *mCRP* markers, whereas *mCRP-EpiScore* was more strongly associated with mortality and *PCGrimAge*, which might point to its potential application in the development of epigenetic markers of ageing. Interestingly, *mCRP-EpiScore* was one of the protein surrogate markers selected in *bAge* to improve biological age estimation on top of *GrimAge*, although with minimal increase in mortality prediction compared to *GrimAge* [22]. *mCRP-GrimAge2* was developed with adjustment for the 1030 CpGs from *GrimAge* [19] and was able to capture a greater amount of plasma CRP variance than other *mCRP* markers. *mCRP-Hillary*, on the contrary, showed the greatest association with BMI but was only weakly associated with mortality. These differences between *mCRP* markers are likely due to different models for CpG prediction, criteria, and inherent differences in shared features such as lifestyle and genetic factors across cohorts. For example, *mCRP-Ligthart* selected CpGs independent of other CpGs in their replication set instead of those showing the strongest correlation with measured CRP and did not test performance in external datasets other than the replication set [14]. In our study, *mCRP* markers derived using elastic net regression [16, 17, 19] generally performed better in association with CRP and health-related traits than earlier markers calculated as a weighted average using EWAS results [14, 15]. A formal comparison of *mCRP* markers derived from four different models: (1) linear model, (2) elastic net regression, (3) Bayesian penalised regression, and (4) principal-component analysis and elastic net regression was carried out by Hillary et al. [17], who concluded that elastic net regression performed slightly better in CRP prediction and associations with health outcomes. Additional studies are warranted to formally assess how the choice of study sample and analytical methods may influence the validity of epigenetic markers such as *mCRP*. Previous studies constructing and evaluating *mCRP* markers did not adjust for WBC proportions [16, 17, 19]. Whether WBC proportions should be adjusted for in epigenomic studies is under debate and should be carried out with caution [45–47]. In the context of our study, WBC proportions constitute part of the response to inflammation [48], hence being a mediator rather than a confounding factor and were not adjusted for in our primary analysis. The findings after adjustment suggest that, except for *mCRP-Ligthart*, the associations for the *mCRP* markers we considered were not primarily driven by a shift in immune cell types.

Our study had some limitations. First, the participants included were generally healthier than the general population [49], which could limit its generalisability. Nevertheless, these participants were a random sample of the controls from six nested case-control studies, so were relatively unselected with respect to the MCCS cohort. We also had limited data on other aspects of ageing such as cognitive function or frailty, which restricted our analyses of ageing-related traits. In addition, although we assessed all *mCRP* markers in an external cohort and compared the results across markers and models, such comparisons did not include formal quantification of statistical variability; hence, they should be interpreted with some caution. There were only two waves of data collection approximately a decade apart, which limited our ability to assess short-term stability of CRP and *mCRP*, with a likely greater influence of age and incidental health conditions.

In conclusion, the associations of CRP markers with health-related traits and their temporal stability were largely marker-dependent, with no consistent superiority of *mCRP* markers over CRP in our study. Although none of the *mCRP* fully represented CRP, those developed using elastic net regression explained a greater amount of the variance in CRP and showed strong associations with health-related traits, which could generate insights into the future development of molecular markers of ageing. Our findings will help establish the clinical utility of *mCRP* markers as potential tools to monitor chronic inflammation and for other precision medicine applications. Future studies are warranted for comparisons of other methylation-based inflammation markers, clarification of *mCRP* marker composition, and development of biologically informed epigenetic predictors for better understanding of the overlap and interplay between age-related inflammatory processes and epigenetic alterations.

## Supplementary Information

Below is the link to the electronic supplementary material.

ESM 1 (43.1 KB DOCX)

## Data Availability

Due to ethical constraints related to the consent of participants, we cannot share the full deidentified dataset. For most participants included in this study, the data are publicly available under controlled access at dbGaP (#phs003213.v1.p1, for which more details can be found at https://www.ncbi.nlm.nih.gov/projects/gap/cgi-bin/study.cgi?study_id=phs003213.v1.p1).
