# Supplementary material for: DNA methylation-based surrogate markers of C-reactive protein and their associations with health-related traits
Source: GeroScience. 2026 Jan 22;48(6):7977–90. doi: 10.1007/s11357-025-02085-8 (PMC13638940; doi:10.1007/s11357-025-02085-8)
Supplement: Supplementary file 1 [file 11357_2025_2085_MOESM1_ESM.docx]

**DNA methylation-based surrogate markers of C-reactive protein and their associations with health-related traits**

**Supplemental Material**

**Supplementary Methods: Covariates**

**Table S1.** The proportion of missing covariates.

**Table S2.** Characteristics of the study sample from the Melbourne Collaborative Cohort study at wave 2 (N=947).

**Table S3.** The concordance (C-index) for plasma hsCRP and *mCRP* markers and *PCGrimAge* (N=947).

**Table S4.** Sensitivity analysis: excluding participants with high plasma hsCRP levels (>10 µg/mL) (N = 907, N deaths = 295).

**Table S5.** Temporal stability of CRP and *mCRP* markers: inter-wave correlations and associations between changes over follow-up and follow-up time (N=899).

**Table S6.** Summary of results for plasma hsCRP and *mCRP* markers.

**Supplementary Methods**

**Covariates**

Data on sociodemographic and lifestyle factors were collected via questionnaires during face-to-face interview at wave 2, including age (years), sex (male, female), country of birth (Australia/New Zealand, Northern Europe, Southern Europe), socioeconomic status (socioeconomic indexes for areas [SEIFA] score (1), based on postcode of residence, decile), education level (1-8, with 1 refers to never attended school, 8 refers to completed tertiary degree or diploma), smoking status (current, former, never smokers), smoking packyears (log-transformed), alcohol consumption (grams/day), physical activity (frequency score, 0-16 (2)), and diet quality (alternative healthy eating index 2010 [AHEI-2010], calculated from the food frequency questionnaire). Total cholesterol levels (mmol/L) was assayed from plasma sample.

The MET score was calculated as the sum of multiplying time spent on each type of physical activity (walking, vigorous, and non-vigorous activities) collected from the International Physical Activity Questionnaire short-form (IPAQ-short) (3) by its corresponding MET value to summarise the weekly frequency, duration, and intensity of physical activities (4, 5).

**References**

1.	Australian Bureau of Statistics A. Socio-economic indexes for areas (SEIFA), Australia. ABS; 2023. (<https://www.abs.gov.au/statistics/people/people-and-communities/socio-economic-indexes-areas-seifa-australia/latest-release>). (Accessed 05/28 2023).

2.	Ainsworth BE, Haskell WL, Leon AS, et al. Compendium of Physical Activities: classification of energy costs of human physical activities. *Medicine & Science in Sports & Exercise* 1993;25(1).

3.	Sjostrom M, Ainsworth BE, Bauman A, et al. Guidelines for data processing analysis of the International Physical Activity Questionnaire (IPAQ) - Short and long forms. 2005.

4.	Jetté M, Sidney K, Blümchen G. Metabolic equivalents (METS) in exercise testing, exercise prescription, and evaluation of functional capacity. *Clinical Cardiology* 1990;13(8):555-65.

5.	Milne RL, Fletcher AS, MacInnis RJ, et al. Cohort Profile: The Melbourne Collaborative Cohort Study (Health 2020). *International Journal of Epidemiology* 2017;46(6):1757-i %U <http://academic.oup.com/ije/article/46/6//3882696>.

**Table S1.** The proportion of missing covariates.

| Variables | N_missing_ | % missing |
| --- | --- | --- |
| SEIFA-10 | 38 | 4.0% |
| Smoking packyears | 13 | 1.4% |
| Alcohol consumption | 50 | 5.3% |
| MET score | 27 | 2.9% |
| AHEI-2010 | 101 | 10.7% |
| BMI | 1 | 0.1% |
| Total cholesterol | 1 | 0.1% |

* BMI = body mass index; AHEI-2010 = Alternative Healthy Eating Index 2010; MET = Metabolic Equivalent of Task.

**Table S2.** Characteristics of the study sample from the Melbourne Collaborative Cohort study at wave 2 (N=947).

|  | **Overall (N=947)** | **Alive**  **(N=628)** | **Dead**  **(N=319)** |
| --- | --- | --- | --- |
| **Age, mean (SD)** | 68.9 (8.0) | 66.3 (7.7) | 74.0 (5.9) |
| **Sex, N (%)** |  |  |  |
| Males | 650 (68.6%) | 413 (65.8%) | 237 (74.3%) |
| Females | 297 (31.4%) | 215 (34.2%) | 82 (25.7%) |
| **Country of birth, N (%)** |  |  |  |
| Australia/New Zealand | 729 (77.0%) | 477 (76.0%) | 252 (79.0%) |
| Northern Europe | 82 (8.7%) | 55 (8.8%) | 27 (8.5%) |
| Southern Europe | 136 (14.4%) | 96 (15.3%) | 40 (12.5%) |
| **SEIFA score, median (IQR)** | 7 (4, 9) | 7 (4, 9) | 6 (4, 8) |
| **Smoking status, N (%)** |  |  |  |
| Current smoker | 50 (5.3%) | 34 (5.4%) | 16 (5.0%) |
| Former smoker | 391 (41.3%) | 234 (37.3%) | 157 (49.2%) |
| Never smoker | 506 (53.4%) | 360 (57.3%) | 146 (45.8%) |
| **Smoking pack-years, mean (SD)** | 2.8 (3.0) | 2.5 (2.9) | 3.5 (3.1) |
| **Alcohol consumption, median (IQR)** | 9.3 (0, 22.7) | 9.9 (0.8, 23.3) | 8.0 (0, 22.2) |
| **Education level, median (IQR)** | 5 (4, 8) | 5 (4, 8) | 4 (4, 6) |
| **BMI (kg/m2), mean (SD)** | 27.1 (4.1) | 27.1 (4.2) | 26.9 (4.1) |
| **AHEI-2010, mean (SD)^a^** | 52.5 (10.2) | 53.0 (10.1) | 51.6 (10.3) |
| **MET score, median (IQR)** | 19.4 (7.4, 34.7) | 19.3 (8.3, 34.7) | 19.5 (6.0, 34.7) |
| **Total cholesterol (mmol/L), mean (SD)** | 5.0 (0.9) | 5.1 (0.9) | 4.8 (1.0) |
| ***PCGrimAge* (age-adjusted), mean (SD)** | 0.14 (3.40) | -0.30 (3.08) | 1.00 (3.81) |
| **Plasma hsCRP (µg/mL), mean (SD)** | 2.23 (3.82) | 1.73 (2.80) | 3.22 (5.16) |
| **lnCRP, mean (SD)** | 0.87 (0.68) | 0.77 (0.60) | 1.06 (0.78) |
| ***mCRP-Ligthart*, mean (SD)** | 0.004 (0.001) | 0.004 (0.001) | 0.004 (0.001) |
| ***mCRP-Wielscher*, mean (SD)** | -22.8 (2.1) | -23.2 (2.0) | -22.1 (2.0) |
| ***mCRP-EpiScore*, mean (SD)** | -0.12 (0.01) | -0.12 (0.01) | -0.12 (0.01) |
| ***mCRP-GrimAge2*, mean (SD)** | 0.27 (0.42) | 0.20 (0.41) | 0.40 (0.42) |
| ***mCRP-Hillary*, mean (SD)** | 0.00 (0.30) | -0.02 (0.29) | 0.05 (0.32) |

*IQR = interquartile range; SD = standard deviation; SEIFA = socioeconomic index for areas; BMI = body mass index; AHEI-2010 = Alternative Healthy Eating Index 2010; MET = Metabolic Equivalent of Task; hsCRP = high-sensitive C-reactive Protein; lnCRP = log-transformed CRP [i.e. ln(CRP+1)].

^a^AHEI-2010 is a literature-based score that can range between 0 and 110; in our sample, it ranges 23-83.

**Table S3.** The concordance (C-index) for plasma hsCRP and *mCRP* markers and *PCGrimAge* (N=947).

| **Markers** | **C-index** | | **Markers** | **C-index** | | **Markers** | **C-index** | |
| --- | --- | --- | --- | --- | --- | --- | --- | --- |
|  | **Unadjusted** | **+ Model 0** |  | **Unadjusted** | **+ Model 0** |  | **Unadjusted** | **+ Model 0** |
| CRP | 0.595 | 0.753 |  |  |  |  |  |  |
| *mCRP-Ligthart* | 0.512 | 0.742 | CRP *+ mCRP-Ligthart* | 0.595 | 0.753 | *mCRP-Ligthart + PCGrimAge* | 0.602 | 0.762 |
| *mCRP-Wielscher* | 0.642 | 0.748 | CRP *+ mCRP-Wielscher* | 0.659 | 0.757 | *mCRP-Wielscher + PCGrimAge* | 0.648 | 0.762 |
| *mCRP-EpiScore* | 0.656 | 0.754 | CRP *+ mCRP-EpiScore* | 0.664 | 0.758 | *mCRP-EpiScore + PCGrimAge* | 0.656 | 0.762 |
| *mCRP-GrimAge2* | 0.618 | 0.750 | CRP *+ mCRP-GrimAge2* | 0.630 | 0.755 | *mCRP-GrimAge2 + PCGrimAge* | 0.639 | 0.762 |
| *mCRP-Hillary* | 0.564 | 0.746 | CRP *+ mCRP-Hillary* | 0.601 | 0.754 | *mCRP-Hillary + PCGrimAge* | 0.605 | 0.762 |
| *PCGrimAge* | 0.602 | 0.762 | CRP *+ PCGrimAge* | 0.623 | 0.765 |  |  |  |
| All five *mCRP* | 0.673 | 0.755 | CRP + all five *mCRP* | 0.680 | 0.759 | All five *mCRP* + *PCGrimAge* | 0.673 | 0.763 |
| Model 0: age+sex+country of birth | 0.742 | - |  |  |  | All six CRP markers + *PCGrimAge* | 0.680 | 0.766 |

*Model 0 refers to the model include only age, sex, and country of birth.

***PCGrimAge* was adjusted for age.

***hsCRP = high-sensitive C-reactive protein. hsCRP was log-transformed as ln(CRP+1).

| Models | | hsCRP | | | *mCRP-Ligthart* | | | *mCRP-Wielscher* | | |
| --- | --- | --- | --- | --- | --- | --- | --- | --- | --- | --- |
|  |  | HR | 95% CI | P | HR | 95% CI | P | HR | 95% CI | P |
| Model 1^a^ | Age, sex, country of birth | 1.27 | 1.13-1.42 | **3×10^-5^** | 1.02 | 0.91-1.15 | 0.74 | 1.24 | 1.09-1.41 | **8×10^-4^** |
| Model 2^b^ | + *PCGrimAge* | 1.22 | 1.09-1.36 | **7×10^-4^** | 1.08 | 0.96-1.22 | 0.18 | 1.06 | 0.92-1.22 | 0.43 |
|  | CRP + *mCRP-Ligthart* | 1.27 | 1.13-1.42 | **3×10^-5^** | 1.02 | 0.91-1.15 | 0.73 |  |  |  |
|  | CRP + *mCRP-Wielscher* | 1.24 | 1.11-1.39 | **2×10^-4^** |  |  |  | 1.20 | 1.06-1.36 | **4×10^-3^** |
|  | CRP + *mCRP-EpiScore* | 1.21 | 1.08-1.36 | **1×10^-3^** |  |  |  |  |  |  |
|  | CRP + *mCRP-GrimAge2* | 1.23 | 1.09-1.38 | **5×10^-4^** |  |  |  |  |  |  |
|  | CRP + *mCRP-Hillary* | 1.26 | 1.12-1.42 | **1×10^-4^** |  |  |  |  |  |  |
| Model 3^c^ | All six markers | 1.23 | 1.09-1.38 | **7×10^-4^** | 1.06 | 0.93-1.21 | 0.36 | 1.13 | 0.97-1.32 | 0.12 |
| Model 4^d^ | Model 1 + lifestyle covariates | 1.25 | 1.11-1.42 | **3×10^-4^** | 1.01 | 0.90-1.14 | 0.85 | 1.18 | 1.03-1.34 | **0.01** |
|  |  | *mCRP-EpiScore* | | | *mCRP-GrimAge2* | | | *mCRP-Hillary* | | |
| Model 1 | Age, sex, country of birth | 1.34 | 1.18-1.52 | **3×10^-6^** | 1.21 | 1.07-1.37 | **3×10^-3^** | 1.09 | 0.97-1.23 | 0.14 |
| Model 2 | + *PCGrimAge* | 1.07 | 0.91-1.26 | 0.39 | 1.08 | 0.95-1.24 | 0.25 | 1.01 | 0.90-1.15 | 0.82 |
|  | CRP + *mCRP-Ligthart* |  |  |  |  |  |  |  |  |  |
|  | CRP + *mCRP-Wielscher* |  |  |  |  |  |  |  |  |  |
|  | CRP + *mCRP-EpiScore* | 1.29 | 1.14-1.47 | **8×10^-5^** |  |  |  |  |  |  |
|  | CRP + *mCRP-GrimAge2* |  |  |  | 1.14 | 1.00-1.30 | **0.04** |  |  |  |
|  | CRP + *mCRP-Hillary* |  |  |  |  |  |  | 1.02 | 0.90-1.16 | 0.73 |
| Model 3 | All six markers | 1.27 | 1.09-1.48 | **3×10^-3^** | 1.07 | 0.92-1.24 | 0.39 | 0.87 | 0.75-1.02 | 0.08 |
| Model 4 | Model 1 + lifestyle covariates | 1.27 | 1.12-1.45 | **2×10^-4^** | 1.16 | 1.02-1.32 | **0.03** | 1.05 | 0.92-1.19 | 0.47 |

**Table S4.** Sensitivity analysis: excluding participants with high plasma hsCRP levels (>10 µg/mL) (N = 907, N deaths = 295).

^a^Model 1 adjusted for age, sex, and country of birth.

^b^Model 2 adjusted for age, sex, country of birth, and age-adjusted *PCGrimAge* or the specified corresponding CRP or *mCRP* markers.

^c^Model 3 included age, sex, country of birth, CRP, and all three *mCRP* markers.

^d^Model 4 adjusted for age, sex, country of birth, SEIFA-10, education levels, smoking status, smoking packyears, alcohol consumption (spline), AHEI-2010, physical activity, and BMI (spline).

*hsCRP = high-sensitive C-reactive protein. hsCRP was log-transformed as ln(CRP+1). CRP, *mCRP*, *PCGrimAge*, and BMI were standardised to a mean of 0 and standard deviation of 1.

**Table S5.** Temporal stability of CRP and *mCRP* markers: inter-wave correlations and associations between changes over follow-up and follow-up time (N=899).

| **Markers** | **Inter-wave correlations** | **Mean (SD)** | | **Associations with follow-up time^*^** | | |
| --- | --- | --- | --- | --- | --- | --- |
|  |  | **Baseline** | **Wave 2** | **β** | **95% CI** | **P** |
| hsCRP^**^ | 0.49 | 0.84 (0.65) | 0.86 (0.68) | 0.04 | -0.02, 0.09 | 0.19 |
| *mCRP-Ligthart* | 0.56 | 0.004 (0.001) | 0.004 (0.001) | -0.04 | -0.09, 0.01 | 0.13 |
| *mCRP-Wielscher* | 0.67 | -24.00 (2.12) | -22.82 (2.06) | 0.03 | -0.02, 0.09 | 0.28 |
| *mCRP-EpiScore* | 0.60 | -0.13 (0.01) | -0.12 (0.01) | 0.13 | 0.08, 0.19 | 3×10^-6^ |
| *mCRP-GrimAge2* | 0.47 | -0.11 (0.46) | 0.26 (0.43) | 0.11 | 0.05, 0.16 | 2×10^-4^ |
| *mCRP-Hillary* | 0.48 | -0.05 (0.24) | -0.002 (0.29) | 0.05 | -0.01, 0.10 | 0.08 |
| *PCGrimAge^***^* | 0.95 | 66.21 (7.12) | 75.99 (7.59) | 0.43 | 0.38, 0.47 | 3×10^-60^ |

^*^ Univariate models were used to assess associations between changes in CRP, *mCRP* and *PCGrimAge* over follow-up (wave 2 value – baseline value) and time between baseline and wave 2 visits. Crude values (not adjusted for age) were used in the analyses. Changes in all markers were standardised to a mean of 0 and standard deviation of 1 in the models assessing their associations with follow-up time.

^**^hsCRP = high-sensitive C-reactive protein. hsCRP was log-transformed as ln(hsCRP+1).

^***^PCGrimAge was used as a reference as it is considered as a powerful epigenetic marker of ageing.

**Table S6.** Summary of results for plasma hsCRP and *mCRP* markers.

| ***Markers*** | **Correlation with CRP** | **Associations with health-related traits** | | | **Temporal stability** |
| --- | --- | --- | --- | --- | --- |
|  |  | **Mortality** | **BMI** | ***PCGrimAge*** |  |
| **hsCRP** | - | Positive, no significant attenuation after adjusting for *mCRP* or *PCGrimAge* | Positive, *attenuated after adjusting for mCRP-Hillary* | Positive, *attenuated after adjusting for mCRP-Episcore* | Relatively stable at lower levels |
| ***mCRP-Ligthart*** | No | Null | Weak positive, no significant changes after adjusting for CRP/*PCGrimAge* | Negative, no significant changes after adjusting for CRP | Higher ICC compared to CRP |
| ***mCRP-Wielscher*** | Weak-to-moderate | Positive, attenuated to null only after adjustment of *PCGrimAge* | Weak positive, attenuated close to null after adjustment of CRP, less so after adjustment of *PCGrimAge* | Positive, minimal attenuation after adjusting for CRP | **The highest ICC** |
| ***mCRP-EpiScore*** | Moderate | **The strongest association**, attenuated to null *only* after adjustment of *PCGrimAge* | Weak positive, attenuated to null after adjusting for CRP, close to null after adjusting for *PCGrimAge* | **The strongest association**, no significant changes after adjusting for CRP | Higher ICC compared to CRP |
| ***mCRP-GrimAge2*** | **Moderate-to-strong** | Positive; attenuated close to null after adjusting for CRP, and to null after adjustment of *PCGrimAge* | Positive **(similar to the association of CRP)**, attenuated after adjusting for CRP | Positive, no significant changes after adjusting for CRP | The lowest ICC |
| ***mCRP-Hillary*** | Moderate | **Weak positive**, attenuated to null after adjustment of CRP/*PCGrimAge* | **The strongest association**, attenuated after adjusting for CRP | Positive, no significant changes after adjusting for CRP | Lower ICC compared to CRP |

*hsCRP = high-sensitive C-reactive protein
